# Local densities and habitat preference of the critically endangered spotted handfish (*Brachionichthys hirsutus*): Large scale field trial of GPS parameterised underwater visual census and diver attached camera

**DOI:** 10.1371/journal.pone.0201518

**Published:** 2018-08-13

**Authors:** Lincoln S. C. Wong, Tim P. Lynch, Neville S. Barrett, Jeffrey T. Wright, Mark A. Green, David J. H. Flynn

**Affiliations:** 1 Institute for Marine and Antarctic Studies, University of Tasmania, Hobart TAS, Australia; 2 CSIRO Ocean and Atmosphere, Hobart TAS, Australia; Sichuan University, CHINA

## Abstract

The critically endangered spotted handfish (*Brachionichthys hirsutus*) is restricted to a limited number of locations in south-eastern Tasmania, Australia. As is often the case for rare species, conducting statistically adequate surveys for *B*. *hirsutus* can be costly and time consuming due to the low probability of encountering individuals. For the first time we used a highly efficient and rigorous Global Positioning System (GPS) parameterised underwater visual census (GUVC) to survey *B*. *hirsutus* abundance within all nine known local populations in the Derwent Estuary within one season. In addition, a benthic microhabitat assessment was conducted simultaneously using a GoPro^®^ camera attached to diver to determine *B*. *hirsutus* microhabitat preferences. *B*. *hirsutus* local populations varied between sites, with densities ranging from 1.58 to 43.0 fishes per hectare. *B*. *hirsutus* demonstrates a strong preference for complex microhabitat features, such as depressions and ripple formations filled with biogenic substrates (e.g. shells) but avoids simple, low relief microhabitats (e.g. sand flats) and areas dominated by ephemeral, filamentous algae. Complex microhabitats may enable *B*. *hirsutus* to avoid predators, increase forage opportunities or provide higher quality spawning sites. This first wide-scale application of GUVC for *B*. *hirsutus* allowed us to survey a larger number of sites than previously possible to provide a robust reference point for future long-term monitoring.

## Introduction

Coastal urbanisation has resulted in significant localised impacts to many marine species through habitat modification, introduction of invasive species and pollution [1–4]. Species with low fecundity, restricted dispersal and small geographic ranges may be more susceptible to these impacts, causing population declines [5]. However, declines in marine species are often poorly understood even when threatening processes are known and this is often due to a lack of baseline and habitat data [6]. As such, effective monitoring of threatened species population and habitat use is key to developing appropriate conservation management. Threatened species, however, can be sparsely distributed, cryptic and with fragmented populations, making monitoring challenging and resource intensive [7]. An added limitation is that surveying threatened species requires non-destructive sampling methods to prevent additional impact to the population [8].

A common non-destructive method for monitoring aquatic species is underwater visual census (UVC) which is typically parameterised by search area or time [9, 10]. Area-based UVC is often based on fixed length transect (50m to 100m) to standardised search area [11, 12], while time-based UVC are not constrained by transect length, resulting in a longer and variable search area which often cannot be precisely quantified [10].

In the last decade, improvements in Global Positioning System (GPS) accuracy has allowed variable length, timed UVC to be improved [13]. By towing a surface float with a GPS unit, geo-information (location, area) can be measured, allowing transects standardisation through comparable units (e.g. density) without the need for underwater guide lines, saving time which can then be used to extend the search area [14–16].

The GPS parameterised UVC (GUVC) method increases search efficiency and therefore presents an effective alternative to conventional methods for surveying coastal threatened species, as robust data can be collected with limited resources. GUVC have been previously used to survey the endangered humphead wrasse (*Cheilinus undulatus*) [17], reef fish assemblages [15] and spotted handfish (*Brachionichthys hirsutus*) [16].

Understanding species-habitat relationships of threatened species can also provide insights for their conservation [18, 19]. Species-habitat relationships have been surveyed using various methods, such as remotely operated vehicle (ROV) [20]; autonomous underwater vehicle (AUV) [21]; and UVC [22]. Commercially available high definition action camera (e.g. GoPro^®^) provided a new low cost option for surveying marine habitats [23–25]. With a smaller profile than conventional camera, GoPro camera can be attached to the diver and operated in combination with GUVC to simultaneously survey populations and habitat for small cryptic species. This combination of techniques divides tasks required for species-habitat studies, where the camera provides recording of the habitat for *post-hoc* analysis, allowing the divers to visually focus on searching for the cryptic species.

The Brachionichthyidae are a family of the order Lophiiformes (anglerfish), endemic to Australian waters [26]. One species, the spotted handfish (*Brachionichthys hirsutus*), was once common in the waterways of south-eastern Tasmania [26, 27], but following a major decline between the 1980’s and early 1990’s [28] is now listed as Critically Endangered [29]. *B*. *hirsutus* occurs on soft sediment at depths from 1-60m, though are more easily sighted between 5 and 15m. Surveys conducted in the last 20 years suggest the population is confined to small pockets in the lower Derwent Estuary and D’Entrecasteaux Channel [26, 29–31].

Like all handfish species, *B*. *hirsutus* have restricted dispersal capability with no planktonic larval stage and limited mobility, demonstrating a “walk-like” movement using their modified fins [29]. During breeding season (September to November), adults lay 60–250 eggs in eggmass attached to small protruding substrates such as stalked ascidians (e.g. *Sycozoa* spp.) [32]. Egg hatches after approximately two months, where hatchlings emerge fully metamorphosed and settle immediately on the benthos [29, 32].

This limited dispersal capability suggests *B*. *hirsutus* may rely on specific and spatially restricted habitats [33], and thus may be particularly susceptible to habitat degradation. The decline in *B*. *hirsutus* population has been speculated to be due to historical (e.g. scallop dredging [34]) and ongoing habitat stressors such as coastal urbanisation, introduction of northern Pacific seastars (*Asterias amurensis*) [29], mooring disturbances [16, 31], and climate change [35].

Since the establishment of recovery plans for *B*. *hirsutus*, intermittent surveys have been conducted across various local populations. Resource constraints, however, limited the number of sites that could be surveyed in any given year leading to an incomplete inter-annual record among the known local populations. Using our more logistically efficient GUVC method we undertook the first yearly cross-sectional baseline census of *B*. *hirsutus* abundance throughout all known local populations in the Derwent estuary. We also undertook the first quantitative microhabitat assessment to determine the species-habitat relationship of *B*. *hirsutus*. We aimed to improve the capacity to monitor and track population trajectories over time and discover if the species had specific habitat needs, in order to enhance our understanding of both threats and the status of the species for more effective management.

## Materials and methods

### Population survey

**Fig 1:**
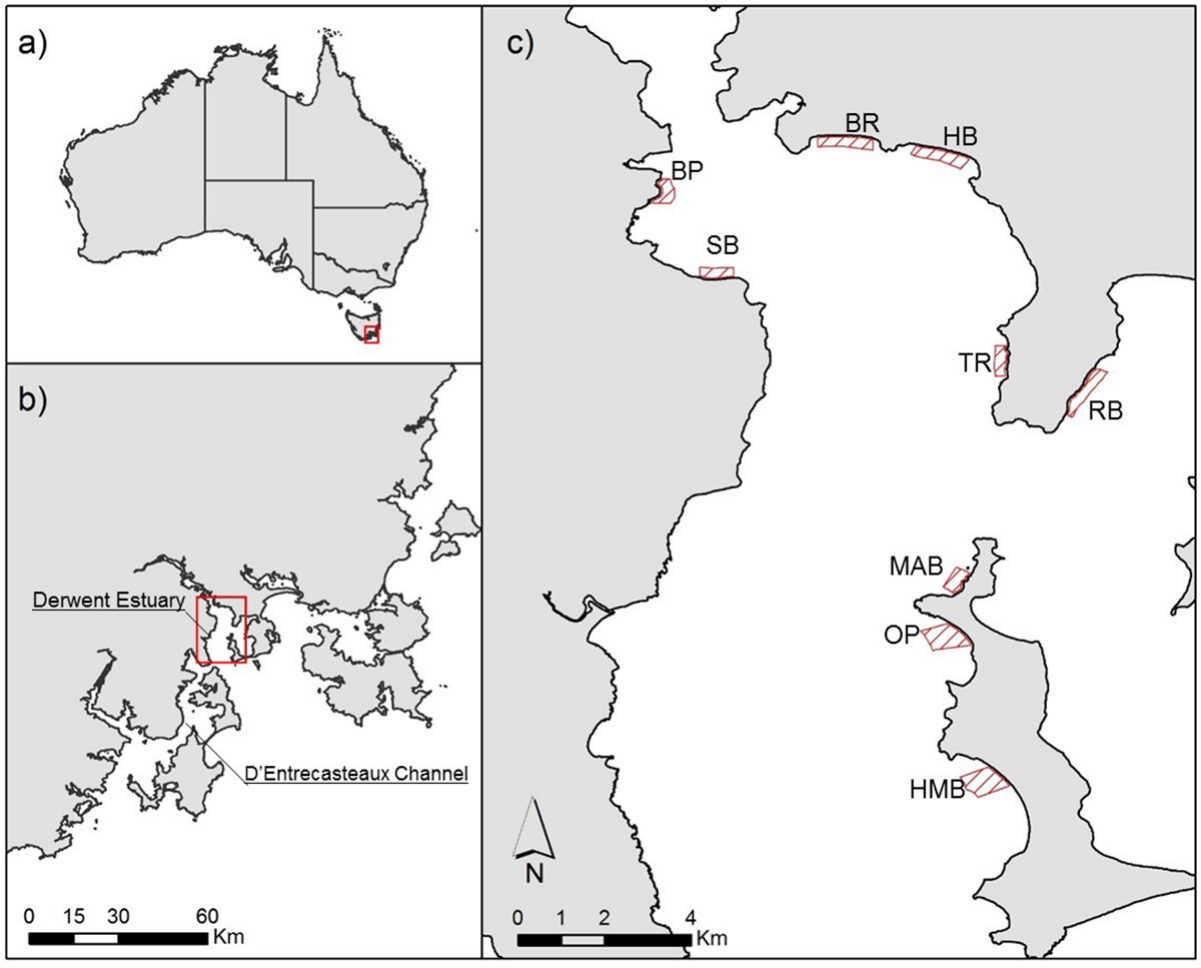
Maps of study regions. (a) Location of South-East Tasmania, (b) the study region in the Derwent Estuary, and (c) the nine Derwent estuary study sites highlighted in red. Abbreviations for each site: Battery Point (BP), Bellerive Beach (BR), Howrah Beach (HB), Half Moon Bay (HMB), Mary-Ann Bay (MAB), Opossum Bay (OP), Ralphs Bay (RB), Tranmere (TR) and Sandy Bay (SB).

Surveys for *B*. *hirsutus* were conducted outside of the breeding season between 23^rd^ April 2015 and 18^th^ August 2015 across all nine sites within the Derwent Estuary known to have local populations (Fig 1). Our survey period only encompassed the non-breeding season of the year, as previous observation suggested *B*. *hirsutus* can have drastic behaviour change including aggregating for breeding [30]. Locations were selected based on searched region of previous surveys by research agencies, university and community groups [e.g.[28, 36–38]]. Dive surveys were conducted weekly throughout the 5 months period, subjected to weather condition. All surveys were conducted under the approval of the University of Tasmania animal ethic committee (permit: A0014803).

**Fig 2:**
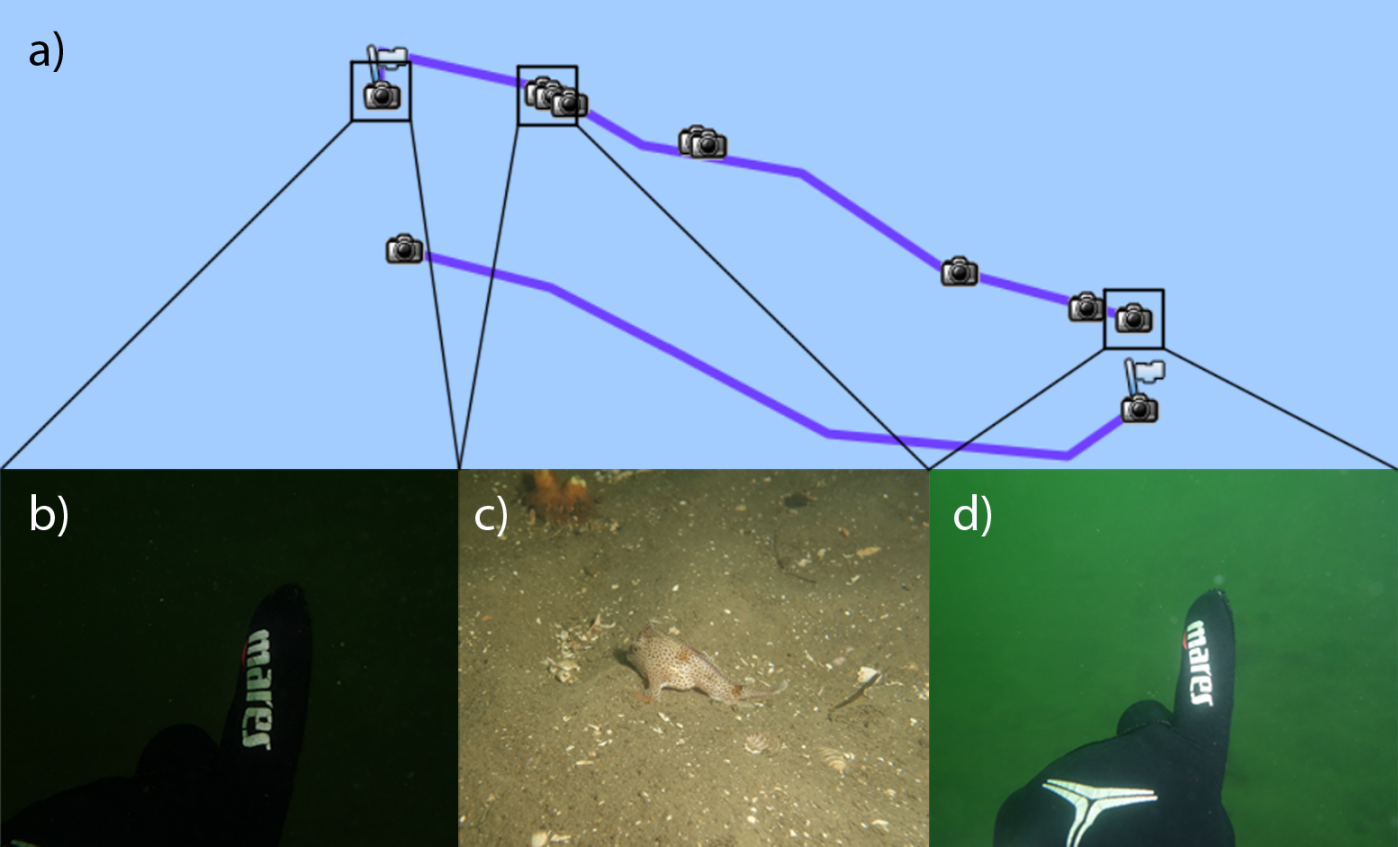
Example of points of interests (POI) captured during each dive. Screen capture of a standard GUVC dive using HOLUX ezTour for Logger. The purple track represented each transect with the camera icon represented geotagged photographs. POI including diver’s signal (b,d) were captured to identified the searched area. In addition, sighting of fish was also recorded using the camera system to identify the location of each sighting (c).

We surveyed *B*. *hirsutus* abundance using a GPS parameterised underwater visual census (GUVC) after Lynch et al [16]. A GPS (HOLUX GPSport 245) was housed in a waterproof case mounted on a surface float and towed by one diver in a two person survey team. A digital camera (Sony RX 100) in an underwater housing was used to photograph points of interests (POI) (Fig 2), including the start and end positions of each transect. These images were timestamped and synchronised by the GPS’s clock.

The starting position for transects were selected from randomly generated coordinates within each site. A total of eight transects were conducted at each site, at depths between 6m and 12m. For this study, we separated each dive (approximately 60 minutes) into two transects, with approximately half the available dive time allocated to each transect. Divers swam abreast approximately at arm’s length apart and 1m above the sediment, navigated parallel to the shoreline. Once the first transect was completed the dive team swam a random number of swim kicks (50–100) to a shallower depth (6-8m) and then commenced the second transect along the back bearing of the initial transect. Divers focused their field of view on the benthos and searched for *B*. *hirsutus* within a swath width of 1.5m for each individual diver. To verify the search width of the survey, divers used the spread of their arms to calibrate the search area throughout the dive.

When *B*. *hirsutus* were located along a transect, a series of photographs were taken to record the habitat and time of the sighting using the GPS calibrated camera. In addition, total length (nearest millimetre) of individual was measured by placing a ruler parallel to the fish. Based on previous observations, individual demonstrated to reach maturity after 2 years, at which total length was over 70mm [32], thus fish smaller than 71mm are classified as juveniles. Previous study [16] modelled the position error for the GPS unit used in this study and found the maximum position error was approximately 13m. To minimise positioning error recorded at each POI, the cable connecting to the GPS float was tightened to ensure the float was directly above the dive team [13, 16].

We used the bundled software (HOLUX ezTour for Logger) to geotag POI photos to extract location data. These POIs were used to extract sections of each dive corresponded to transects. We then computed the swath area of each transect (transect length × swath width) and calculated the density of *B*. *hirsutus* based on the swath area and number of individuals (Eq 1).

$${\hat{D}}_{ij}=\left(n_{ij}/a_{ij}\right)\times 10^{4}$$ (1)

${\hat{D}}_{ij}$: *B*. *hirsutus* density (fishes per hectare) estimate for transect *i* at site *j*

n_ij_: Total fish sighted in transect *i* at site *j*

a_ij_: Swath area (m^2^) of transect *i* at site *j*

We compared *B*. *hirsutus* abundance between sites using a Poisson regression [39, 40]. To account for the variation between transects, we adjusted the swath area of transect with a log link function. We then compared all sites with a pairwise comparison of mean count. Based on the model, we then extrapolated the average density for each site as a standardised unit for comparison (Eq 1).

To evaluate if the current sampling intensity was adequate for future monitoring surveys, we conducted a power analysis on various sample sizes for detecting density changes. We resampled the 2015 dataset without replacement for n^th^ times, where n represented the number of transect conducted within each subpopulation. We pooled density estimates ($\hat{D}$) across all surveyed sites for resampling to account for all variation recorded. We then calculated the mean density for each simulated survey using Eq 1. We calculated the power for detecting scenarios of 25%, 50%, and 75% changes in *B*. *hirsutus* density with a sample size of 8 to 30 transects. To determine the average power for each sample size, we repeated the simulation for 1500 times and determined the mean power level (±SD).

### Microhabitat analysis

We examined benthic microhabitat features to identify the habitat preference of *B*. *hirsutus*. Due to the small scale of the fish, divers were required to position themselves close to the benthos (0.5 to 1m above the bottom) for the survey, our habitat survey only focused on small scale features (< 1m). Action cameras in water-proof housings (GoPro Hero3+, Hero4) were used to capture video data along each transect. The miniaturised camera was secured to the waist strap of one diver’s buoyancy compensator, allowing for continuous, hands-free recording of the benthic habitat without obstruction to the diver’s field of view. This setup allowed the dive team to actively search for fish while still obtaining footages for microhabitat assessment.

A synchronisation procedure between the GoPro cameras and the handheld camera was required as GoPro cameras do not provide a timestamp overlay for footages, prohibiting the imagery to be georeferenced with the GPS track. Prior to entering the water, with the GoPro camera recording and facing the timestamped camera (Sony RX 100), a photo was taken to create a visual reference point on both cameras. By reviewing the elapse time of the GoPro footages where the other camera was present, the video was cross-referenced with the time from the timestamped image. Elapse time of video can be back calculated using the reference point and was geotagged along each transect to provide microhabitat data corresponding to search locations.

**Fig 3:**
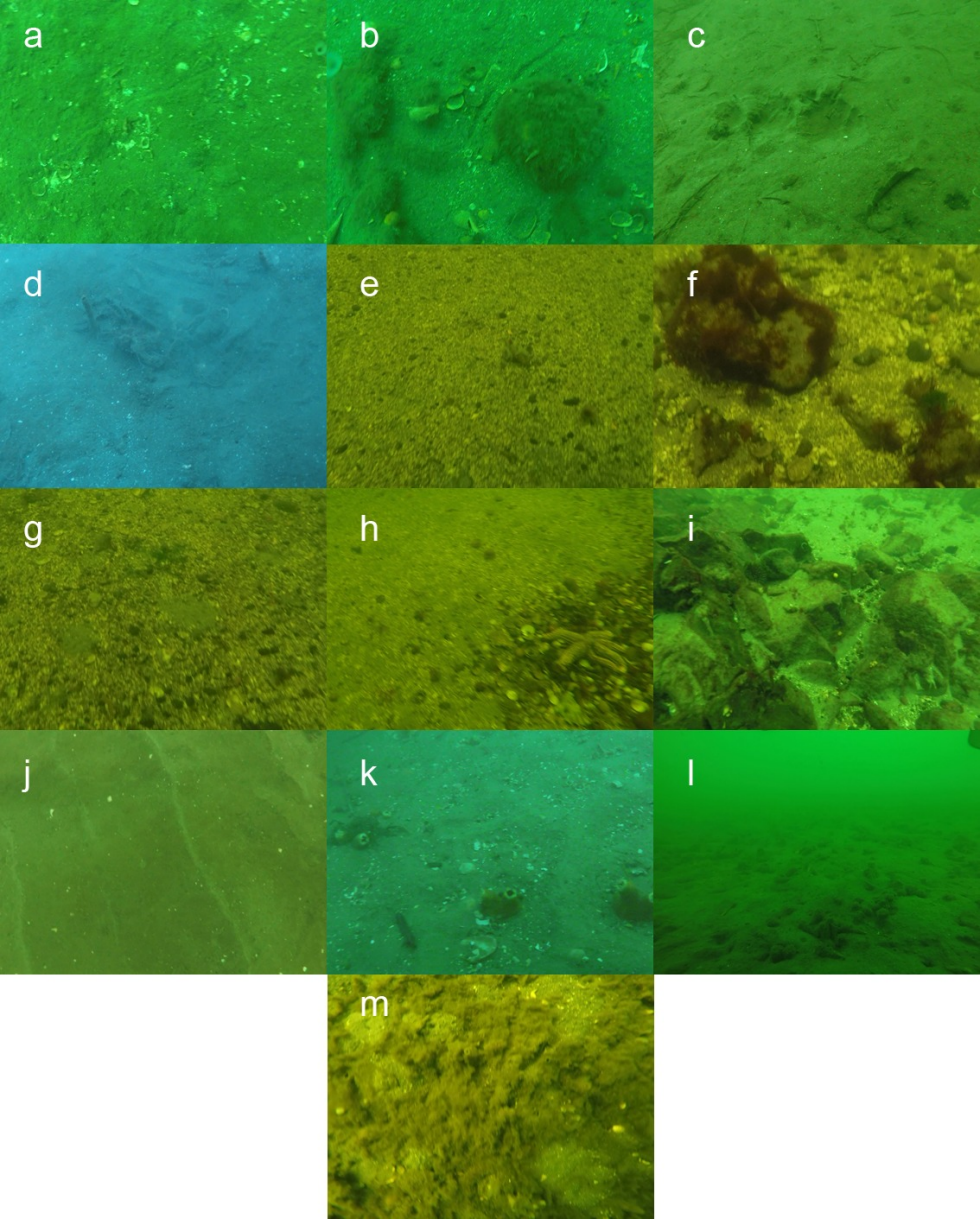
Example of each defined microhabitat features. Letter denote the corresponding microhabitat class in Table 1.

To compare the microhabitat recorded from the video and the still images of microhabitat taken during fish encounter, we extracted still images from the video with a fixed window, whole image approach [41]. A single frame was selected every 15 second to prevent duplicated counting. Extracted frames were then examined, with total count of each microhabitat class summarised for each site. Initially our microhabitat classification was based on the Collaborative and Annotation Tools for Analysis of Marine Imagery and Video (CATAMI) (catami.org) classification scheme. However, following initial field observations, survey areas were predominately flat with limited biota, we therefore described the benthic habitat of all sites into 13 classes with unique operational definition (Table 1, Fig 3), focusing on the fine scale geophysical features to better identify microhabitat variations. All classification was done by one author (LW), to eliminate potential inter-observer bias.

**Table 1:** Operational definition of microhabitat features found across all surveyed *B*. *hirsutus* sites.

Abbreviation	Substrate class	Description	Figure reference
SF	Unconsolidated sand flat	Soft sediment habitat with no identifiable geographical feature	a
SS	Unconsolidated sand flat with low profile structures	Independent 3D structure or erected object visible on sand flat (e.g. cobble, bottle)	b
SD	Unconsolidated sand flat with depression	Indentation formed along the sandflat. No material present within the depression	c
SDF	Unconsolidated sand flat with filled depression	Indentation formed along the sandflat. Material (e.g. shell hash, debris, vegetation) accumulated within the depression	d
GF	Unconsolidated gravel/sand flat	Coarse grain sediment flat with no identifiable geographical feature. Particle are visibly distinguishable	e
GS	Unconsolidated gravel/sand flat with low profile structures	Coarse grain sediment flat with identifiable 3D structure or erected object	f
GD	Unconsolidated gravel/sand flat with depression	Coarse grain sediment flat with indentation formation. Formation are new, with no material deposited in depression	g
GDF	Unconsolidated gravel/sand flat with filled depression	Indentation formed along the coarse grain sediment flat. Material (e.g. shell hash, debris, vegetation) accumulated within the depression	h
R	Rocky reef	Hard consolidated substrate field	i
SR	Sand ripple	Sand ripple formed from current movement. Habitat have a wave formation, with continuous feature across the region	j
SRF	Sand ripple with filled troughs	Sand ripple with material and biota deposited at the troughs of ripple	k
BG	Biogenic gravel/mat (e.g. mussel shells)	Soft sediment habitat with high density of biogenic material such as mussel shells and screw shells.	l
V	Benthic vegetation cover	Soft sediment dominated by vegetation (unspecified) coverage	m

We used the microhabitat sampled across the studied regions to construct a model of expected fish sighting for each microhabitat if fish were randomly distributed and compared this to the observed habitat type associated with sightings of *B*. *hirsutus*. Due to the low number of fish observed at several sites (1 sighting at BR and 3 at RB, TR respectively), we weren’t able to compare habitat preference on the site level. Consequently, all individuals and microhabitat data were pooled across sites to undertake our comparison. We determined the microhabitat preference of *B*. *hirsutus* based on the χ^2^ goodness-of-fit residual between the expected and observed *B*. *hirsutus* sightings at each microhabitat type using Eq 2 [21].

$$PI_{i}=O_{i}-E_{i}$$ (2)

*PI*_*i*_: preference index for habitat *i*

*O*_*i*_: observed frequency of habitat *i* associated with *B*. *hirsutus* distribution

*E*_*i*_: expected frequency of habitat *i* based on the habitat baseline

## Results

**Fig 4:**
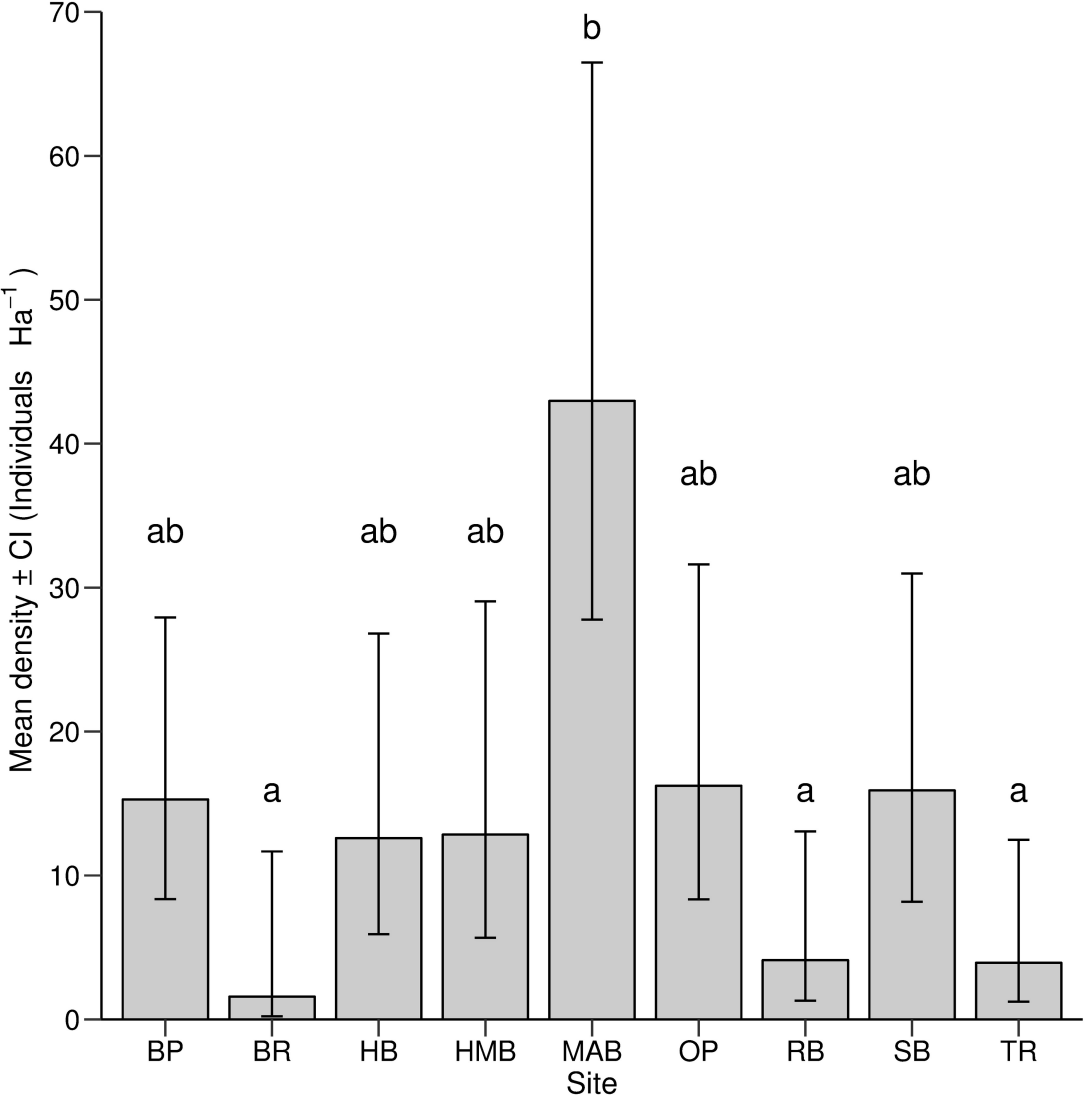
B. hirsutus density by site. *B*. *hirsutus* density (mean ± CI fishes Ha^-1^) estimated at each site. Letters above each bar represented significant grouping of data based on post-hoc pairwise analysis (Tukey HSD test) of transformed data. Standard error for Bellerive (BR) was not presented as only a single fish was found. Site abbreviations as in Fig 1.

A total of 70 observations of *B*. *hirsutus* were made, with fish present at all nine sites. The number of fish observed within local population ranged from one at Bellerive (BR) to 21 at Mary Ann Bay (MAB). The modelled *B*. *hirsutus* density varied between 1.58 fish Ha^-1^ and 43.0 fish Ha^-1^ with significant variation recorded between sites (*χ* ^2^ = 44.9, df = 8, 63, P< 0.001) (Fig 4). Pairwise comparisons indicated differences between density at MAB and the three lowest density sites: Bellerive (BR) (P = 0.0293), Ralph Bay (RB), and Tranmere (TR) (both P< 0.01). Over half of the surveyed sites (n = 5), including Battery Point (BP), Howrah Beach (HB), Half Moon Bay (HMB), Opossum Bay (OP) and Sandy Bay (SB) have a medium density level, with fish densities varied between 12.6 and 16.2 fishes Ha^-1^ (Fig 4). Our model, however were not able to distinguish significant differences from these sites with either low or high density groups.

We recorded the total length of 66 fishes. Measurement was not available for four individuals due to escaping (n = 3) and strike from sand flathead (*Platycephalus bassensis*) (n = 1). Length of fish ranged between 49mm and 120mm with a median length of 83mm. We observed 14 juveniles (<71mm) across five sites (HMB, MAB, OP, SB, TR). While the most juvenile were observed at MAB (n = 6), the highest proportion of juveniles was recorded at HMB, contributing to two-thirds of encounters (n = 4).

**Fig 5:**
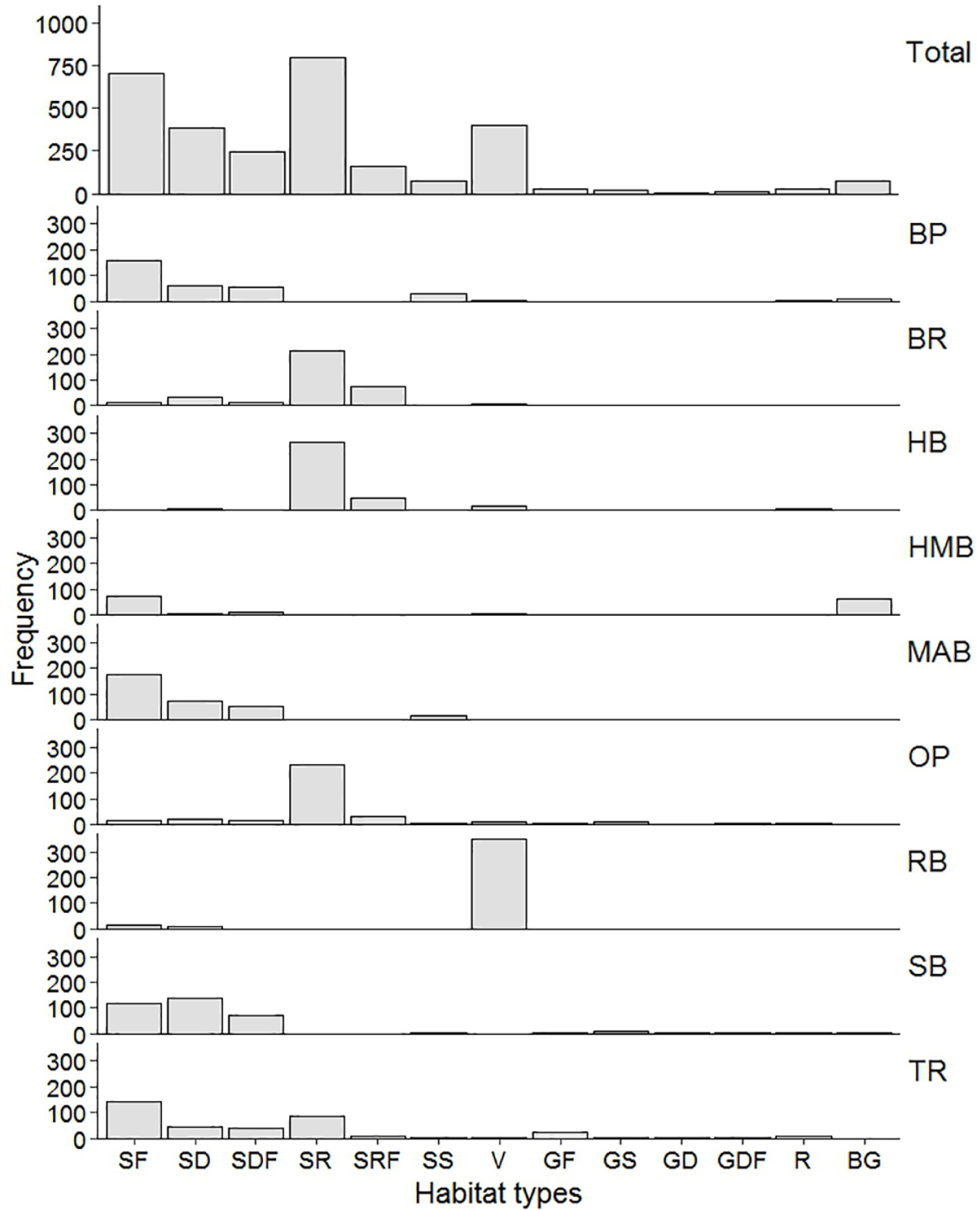
Frequency of microhabitat features by site. Total count of each microhabitat feature classes at each site. Microhabitat feature classes and site abbreviations as in Table 1 and Fig 1.

Due to logistical constraints (e.g. camera failures), microhabitat video were only extracted from four transects at each sites. We extracted a total of 2933 frames from 36 transects and compared to habitat photos from 64 fish encounters. Sites had different compositions of microhabitat features (*χ*^*2*^ = 5700, df = 96, *P*< 0.001; Fig 5). Four sites (BP, HMB, MAB, TR) were dominated by unconsolidated sand flat (SF) with between 38.3% (TR) and 55.4% (MAB) of observations. Similarly, unconsolidated sand ripple (SR) was the most abundant microhabitat at three sites (BR, HB, OP) with 59.2% (BR) to 75.9% (HB) of all extracted frames. Unconsolidated sand flat with empty depression (SD) was observed to be the most common at Sandy Bay (SB) (35%). While Ralphs Bay (RB) was dominated by a vegetation covered substrate (V) (75.1%) of ephemeral, filamentous algae.

**Fig 6:**
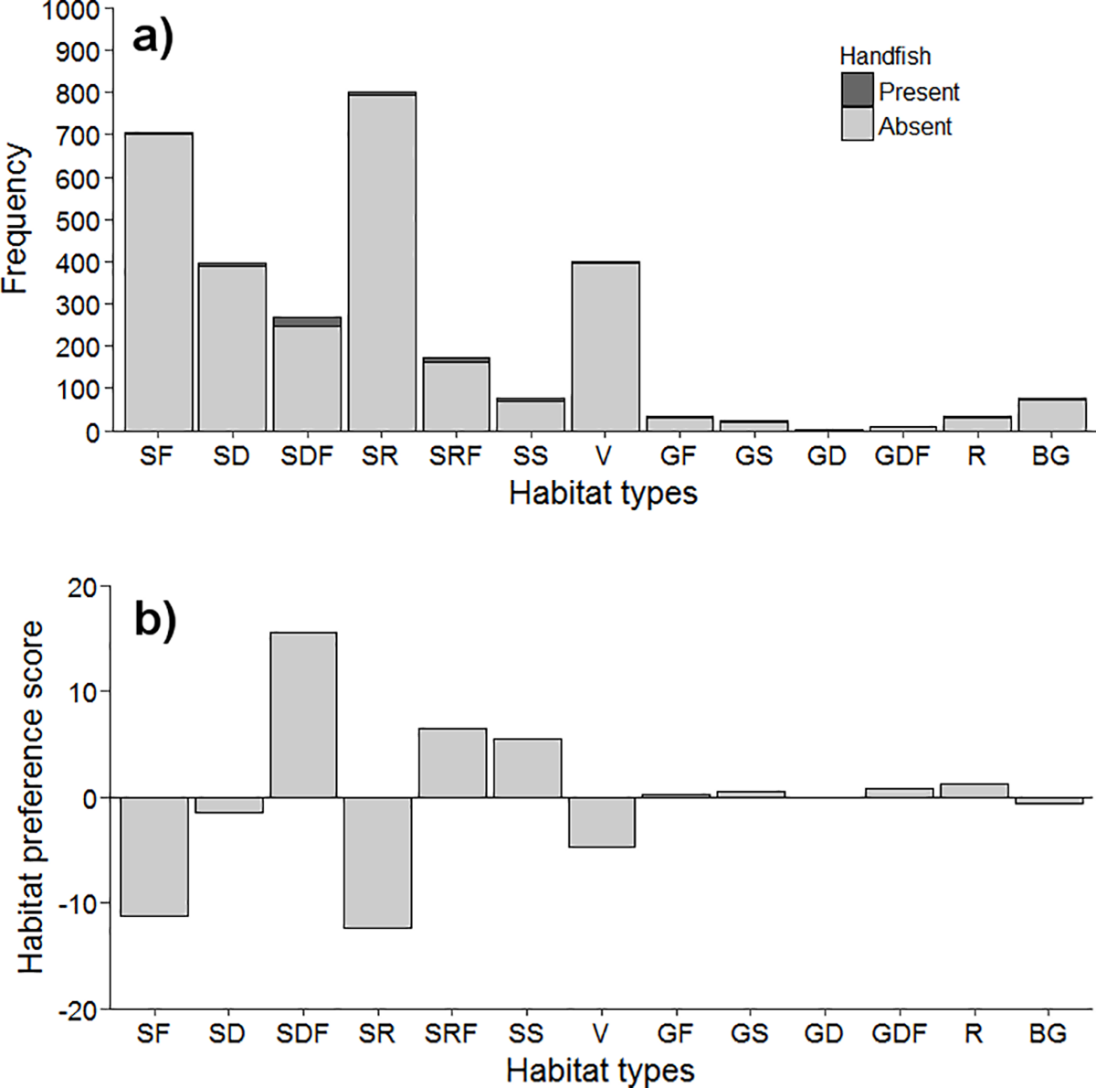
Habitat preference of spotted handfish. (a) Frequency of microhabitat features where B. hirsutus were absent (light grey) and present (dark grey). (b) Habitat preference score (observed proportion—expected proportion) of B. hirsutus for each microhabitat feature class. Microhabitat class abbreviations as in Table 1.

**Fig 7:**
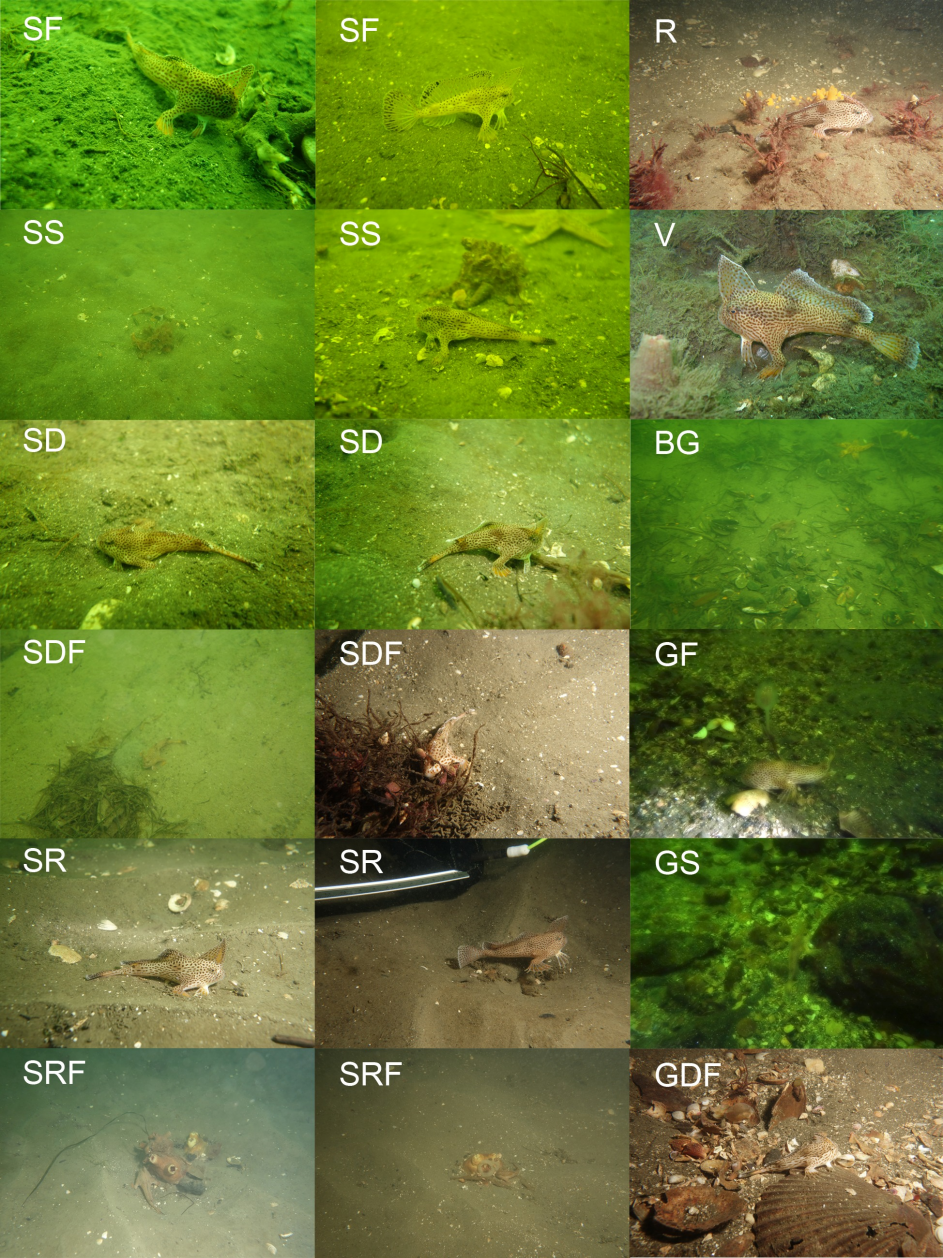
Spotted handfish, habitat relationship observations. Photos of *B*. *hirsutus* observed near each defined microhabitat. The letter denoted the abbreviation for each feature: SF-sand flat; SS-sand flat with structures; SD-sand flat with depression; SDF-sand flat with filled depression; SR-sand ripple; SRF-filled sand ripple; R-rocky reef; V-vegetation mat; BG-Biogenic mat; GF-gravel flat; GS-gravel flat with structures; GDF-gravel flat with depression.

*B*. *hirsutus* were observed on all substrate types except unconsolidated gravel/sand flat with depression (GD) but showed a strong preference for specific substrate types (*χ*^*2*^ = 102, df = 12, *P*< 0.001; Fig 6). The highest number of individuals were sighted in close proximity to complex microhabitats including sand flat with filled depression (SDF, n = 21) and filled sand ripple (SRF, n = 10), which accounted for almost half (48.4%) of all sightings (Fig 5). Fishes were commonly found near depressions/ripples accumulated with shell/shell hash, detritus, polychaete tubes and vegetation (Fig 7). In addition to SDF and SRF, sand flat with low profile structures (SS) (e.g. small boulders) also demonstrated a high habitat score (positive residual) as microhabitat preferred by *B*. *hirsutus* (Fig 6). In contrast, *B*. *hirsutus* demonstrated a strong negative preference for simple microhabitats (SF, SR, V) despite their high availability across all site (Fig 6).

**Fig 8:**
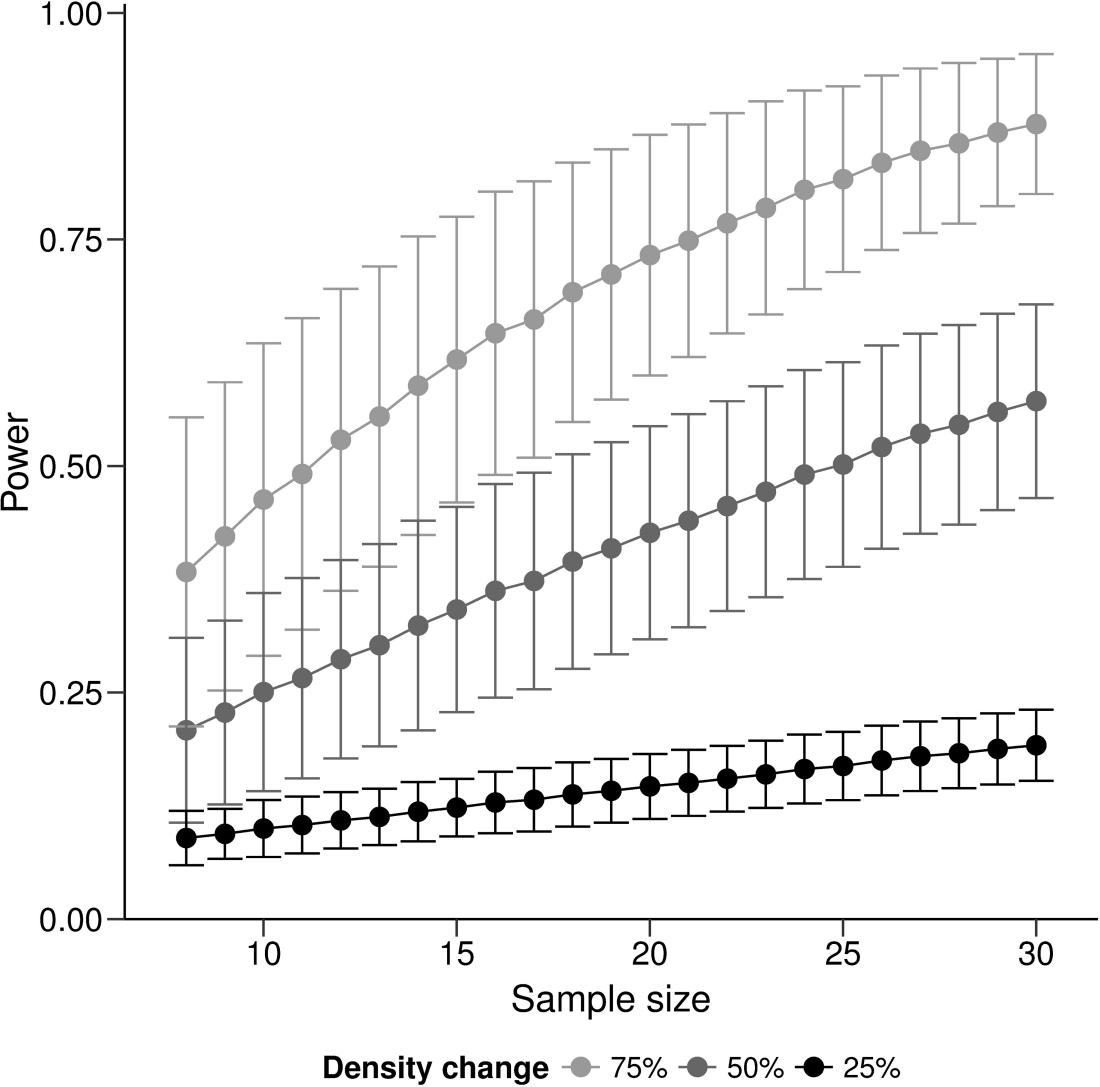
Spotted handfish survey power analysis. Average (±SD) power of detecting *B*. *hirsutus* density changes over time with different sample size (number of GUVC transects) simulated from the 2015 dataset. Analysis were conducted at three different level of changes (light grey– 75%; dark grey– 50%; black– 25%).

Power analysis based on simulation of GUVC surveys indicated the highest sampling power in scenarios with larger (75% or 50%) changes in density (Fig 8). The sharpest increase in power was recorded when sample size increased from 9 to 10 transects, with 10% increase in power for detecting 50% and 75% changes and 7% for detecting 25% density changes. Increased sample size demonstrated small effect on detecting smaller changes (25%), where power only increased from 0.1 (n = 8) to 0.2 (n = 30).

## Discussion

Observed *B*. *hirsutus* densities were similar to historic surveys with most local populations have a density between 10 and 20 fishes Ha^-1^. The highest density site in the present study (MAB), demonstrated a similar density level to only one other historic record at Ralph Bay in 2005 [38]. The spatial variability of densities suggested local population can be affected by microhabitat availability, susceptible to changes to habitat quality such as microhabitat shifts/ degradation, invasive species distribution [5] or availability of spawning habitat.

The strong relationship between *B*. *hirsutus* and microhabitat features quantified previous speculation that individual *B*. *hirsutus* target specific habitat features [42]. Habitat selection by fish species is well documented [20, 43–46], with heterogeneity and complexity often being important factors determining species distribution [47]. The highest proportion of *B*. *hirsutus* were associated with filled depressions/ ripples (SDF, SRF) suggested individuals may be using these features for camouflage and cover, similar to other demersal fish [20, 46]. In the predominately flat sandy areas, features such as depressions and ripples may help break the outline of *B*. *hirsutus*, as does the species spot pattern, which is well suited for camouflaging near shell hash and detritus. The behavioural use of habitat for cover and camouflage may decrease detection rates of *B*. *hirsutus* by visual predators. In soft sediment system, microhabitat features can be dynamic, where features like depressions and ripples can be ephemeral and will be created, refilled or shifted over time [20]. Thus, spatial and temporal variability in the availability of microhabitat features may influence the distribution and density of *B*. *hirsutus* local populations.

As *B*. *hirsutus* demonstrated direct recruitment strategy and limited movement, replenishment may be reliant on self-recruitment within local populations [48]. Through direct recruitment, species can utilise more favourable microhabitats and maximise survival of eggs/ hatchlings [49]. Anecdotal observations indicated B. hirsutus will form aggregations during the breeding season[[30] Barrett, Green pers. comm.], this behaviour may be related to individual’s selection for better habitat and spawning conditions [50]. We only observed low numbers of juveniles, restricted to five specific local populations, suggesting that recruitment and post-hatch survival rate of *B*. *hirsutus* can be spatially variable. Low density and limited movement ranges [51] of individual may also reduce *B*. *hirsutus* encounter rates during the breeding season, decreasing the probability of mating or forcing adults to increase their search effort to locate mates [52, 53], thus potentially increasing the risk of Allee effects on the population [54]. However, despite the spatial scale of this study covering all known local populations of *B*. *hrisutus*, caution is needed when interpreting the data on the population connectivity, as movement and reproductive behaviour of *B*. *hirsutus* still warranted further investigation.

During our survey, we observed a high abundance of ephemeral algae, particularly at Ralphs Bay, similar to another recent survey [38], where drifted algae was observed at Ralphs Bay and Sandy Bay. While drift algae generally persist for a short period of time, algal coverage can alter benthic assemblages in normally bare sediment [55]. The low densities of *B*. *hirsutus* at algae covered area may be due to habitat alteration with ephemeral algae covering the unconsolidated sand habitat which reduced the availability of food, refugia and spawning substrates. Due to the natural variation within the estuary, such as less oceanic influence on the eastern shore [56] can also influence the overall distribution of the population.

With a need for specific spawning substrates [29], *B*. *hirsutus* breeding may only be successful if benthic biota (e.g. stalked ascidian, *Sycozoa* spp.; sponges; seagrass shoot) are abundant within the local population [38]. The introduction and persistence of *A*. *amurensis* within the estuary may have a strong implication on the success of the *B*. *hirsutus* population. As they are an opportunistic benthic predator, *A*. *amurensis* may increase consumption of key epifauna which provided spawning habitat and reduce site complexity [57, 58].

This strong association of *B*. *hirsutus* with complex microhabitats highlights how modification to benthic habitat can have a potential impact on *B*. *hirsutus* populations. Reductions in habitat heterogeneity can reduce refugia and increase the predation rate on demersal fish species [59, 60]. While each local population can be subjected to variable sources of disturbance, their behaviour and life histories suggested populations, particularly at sites with lower density (BR, RB, TR) may be vulnerable to stochastic events [61]. Boat moorings are common infrastructure in coastal region, where currently mooring lease are at full capacity within metropolitan area of the estuary [62]. The mechanical disturbance from common swing mooring can impact the required microhabitat for *B*. *hirsutus*, and has been identified as one of the major threats to *B*. *hirsutes* [31]. One suggested strategy was to study the feasibility and replacement of swing mooring in critical *B*. *hirsutus* site (e.g. Battery Point) to eco-mooring system [63].

With the additional sampling power provide by GUVC compared to fixed length UVC [16], we could, within our resource constraint, sample a wider area, while still providing enough replications to distinguish highly variable sites through this cross-sectional survey. For this study, we covered a total area of 54771m^2^ (5.48 Ha) across all sites with 72 GUVC transects with mean length of 250m (±7.64m SE). To cover the same amount of area using standard UVC with 100m setline, it will require a 54.2% increase to 183 transects. The increased search area per transect also allowed the dataset to be analysed with a more descriptive model, providing more information on the population than a binary response [16].

The ability to detect change is fundamental for long-term monitoring of the conservation status of species. With GUVC we were able to survey all nine sites and distinguish difference between high and low density sites, though we were not able to separate these from medium density sites. We hypothesised this could be due to the increased variance from transects with 0 counts, causing a type II error. Our power simulation, however, suggests that with a slight increase in sample size from 8 to 10 transect the GUVC method would be beneficial for detecting larger scale density changes.

GUVC may therefore not only be effective for surveying cryptic or endangered species and sparse populations, more generally it can also be useful for survey designs or experiments which require survey over a larger spatial scale. However, caution will be needed when incorporating GUVC search in deeper water, as the catenary action of the extended cable between diver and the surface float can affect the accuracy of tracking and positioning each search [14]. In addition, dive time can be limited, hence restricting the maximum searched distance when conducting search in deeper water without highly specific training (e.g. decompression diving and saturation diving), thus limiting the effect of GUVC.

Effective conservation of threatened species requires accurate and up-to-date population data [64, 65]. Through the use of the GUVC method, we successfully implemented the first temporally comparable large scale population survey of *B*. *hirsutus* within the Derwent Estuary. Our results provide the most consistent and robust dataset on *B*. *hirsutus* local populations across a single season collected to date. This study provided the base-line for a long-term monitoring of *B*. *hirsutus* population as part of the ongoing conservation effort [63]. To ensure data are available for tracking *B*. *hirsutus* population health, a robust monitoring regime is required, and all known local population should be monitored over multiple consecutive years. Our habitat survey also provided updated detail on the habitat preference of the species, highlighting the necessity for reducing anthropogenic impact including boat mooring in potential handfish habitat, and using habitat information to guide restoration program such as the deployment of artificial spawning habitat [63].

## References

[1] Edgar GJ, Barrett NS, Graddon DJ, Last PR. The conservation significance of estuaries: a classification of Tasmanian estuaries using ecological, physical and demographic attributes as a case study. Biol Conserv. 2000;92(3):383–97. 10.1016/S0006-3207(99)00111-1 PubMed PMID: WOS:000085249000012.

[2] Halpern BS, Walbridge S, Selkoe KA, Kappel CV, Micheli F, D'Agrosa C, et al A global map of human impact on marine ecosystems. Science. 2008;319(5865):948–52. 10.1126/science.1149345 . 18276889

[3] Jackson JB. Ecological extinction and evolution in the brave new ocean. P Natl Acad Sci. 2008;105(Supplement 1):11458–65. 10.1073/pnas.0802812105 18695220 PMC2556419

[4] Lotze HK, Lenihan HS, Bourque BJ, Bradbury RH, Cooke RG, Kay MC, et al Depletion, degradation, and recovery potential of estuaries and coastal seas. Science. 2006;312(5781):1806–9. 10.1126/science.1128035 . 16794081

[5] Roberts CM, Hawkins JP. Extinction risk in the sea. Trends in Ecology & Evolution. 1999;14(6):241–6. 10.1016/S0169-5347(98)01584-5 10354629

[6] Edgar GJ, Samson CR, Barrett NS. Species extinction in the marine environment: Tasmania as a regional example of overlooked losses in biodiversity. Conserv Biol. 2005;19(4):1294–300. 10.1111/j.1523-1739.2005.00159.x PubMed PMID: WOS:000231118600035.

[7] Chadès I, McDonald-Madden E, McCarthy MA, Wintle B, Linkie M, Possingham HP. When to stop managing or surveying cryptic threatened species. P Natl Acad Sci. 2008;105(37):13936–40. 10.1073/pnas.0805265105 18779594 PMC2544557

[8] Edgar GJ, Stuart-Smith RD, Cooper A, Jacques M, Valentine J. New opportunities for conservation of handfishes (Family Brachionichthyidae) and other inconspicuous and threatened marine species through citizen science. Biol Conserv. 2017;208:174–82. 10.1016/j.biocon.2016.07.028 PubMed PMID: WOS:000399859000020.

[9] Mapstone BD, Ayling AM. An Investigation of Optimum Methods and Unit Sizes for the Visual Estimation of Abundances of Some Coral Reef Organisms. Townsville: Great Barrier Reef Marine Park Authority, 1998.

[10] Murphy HM, Jenkins GP. Observational methods used in marine spatial monitoring of fishes and associated habitats: a review. Mar Freshwater Res. 2010;61(2):236–52. 10.1071/Mf09068 PubMed PMID: WOS:000274913200012.

[11] Edgar GJ, Barrett NS, Morton AJ. Biases associated with the use of underwater visual census techniques to quantify the density and size-structure of fish populations. J Exp Mar Biol Ecol. 2004;308(2):269–90. 10.1016/j.jembe.2004.03.004 PubMed PMID: WOS:000223389900008.

[12] McCormick MI, Choat JH. Estimating total abundance of a large temperate-reef fish using visual strip-transects. Marine Biology. 1987;96(4):469–78. 10.1007/bf00397964

[13] Niedzwiedz G, Schories D. Advances using diver-towed GPS receivers In: Hsueh YH, editor. Global Positioning Systems: Signal Structure, Applications and Sources of Error and Biases. New York: Nova Science Publisher, Inc.; 2013 p. 155–86.

[14] Beck HJ, Feary DA, Figueira WF, Booth DJ. Assessing range shifts of tropical reef fishes: a comparison of belt transect and roaming underwater visual census methods. B Mar Sci. 2014;90(2):705–21. 10.5343/bms.2013.1055 PubMed PMID: WOS:000334975900012.

[15] Lynch T, Green M, Davies C. Diver towed GPS to estimate densities of a critically endangered fish. Biol Conserv. 2015;191:700–6. 10.1016/j.biocon.2015.08.009 PubMed PMID: S0006320715300598.

[16] Schories D, Niedzwiedz G. Precision, accuracy, and application of diver-towed underwater GPS receivers. Environ Monit Assess. 2012;184(4):2359–72. 10.1007/s10661-011-2122-7 . 21614620

[17] Colin PL, Donaldson TJ<, Martin LE, editors. GPS Density Surveys: A New Method for Quantitatively Assessing Reef Fish Spawning Aggregations (and other populations of reef fishes). Seventh Indo-Pacific Fish Conference; 2005; Taipei.

[18] Kovalenko KE, Thomaz SM, Warfe DM. Habitat complexity: approaches and future directions. Hydrobiologia. 2012;685(1):1–17. 10.1007/s10750-011-0974-z PubMed PMID: WOS:000300673500001.

[19] Morris DW. Toward an ecological synthesis: a case for habitat selection. Oecologia. 2003;136(1):1–13. 10.1007/s00442-003-1241-4 12690550

[20] Auster PJ, Malatesta RJ, Larosa SC. Patterns of Microhabitat Utilization by Mobile Megafauna on the Southern New-England (USA) Continental-Shelf and Slope. Mar Ecol Prog Ser. 1995;127(1–3):77–85. 10.3354/meps127077 PubMed PMID: WOS:A1995TG88000008.

[21] Seiler J, Williams A, Barrett N. Assessing size, abundance and habitat preferences of the Ocean Perch Helicolenus percoides using a AUV-borne stereo camera system. Fish Res. 2012;129(0):64–72. 10.1016/j.fishres.2012.06.011 PubMed PMID: WOS:000308057800009.

[22] Alexander TJ, Barrett N, Haddon M, Edgar G. Relationships between mobile macroinvertebrates and reef structure in a temperate marine reserve. Mar Ecol Prog Ser. 2009;389:31–44. 10.3354/meps08210 PubMed PMID: WOS:000270673100003.

[23] Gintert B, Gleason ACR, Cantwell K, Gracias N, Gonzalez M, Pamela Reid R, editors. Third-Generation Underwater Landscape Mosaics for Coral Reef Mapping and Monitoring. Proceedings of the 12th International Coral Reef Symposium; 2012; Cairns.

[24] Schmidt VE, Rzhanov Y, editors. Measurement of micro-bathymetry with a GOPRO underwater stereo camera pair. 2012 Oceans; 2012 14–19 Oct. 2012; Hampton Roads, VA, USA.

[25] Struthers DP, Danylchuk AJ, Wilson ADM, Cooke SJ. Action Cameras: Bringing Aquatic and Fisheries Research into View. Fisheries. 2015;40(10):502–12. 10.1080/03632415.2015.1082472 PubMed PMID: WOS:000362637000005.

[26] Last PR, Gledhill DC. A revision of the Australian handfishes (Lophiiformes: Brachionichthyidae), with descriptions of three new genera and nine new species. Zootaxa. 2009;(2252):1–77. PubMed PMID: WOS:000270565600001.

[27] Last PR, Gledhill DC, Holmes BH. A new handfish, Brachionichthys australis sp nov (Lophiiformes: Brachionichthyidae), with a redescription of the critically endangered spotted handfish, B-hirsutus (Lacepede). Zootaxa. 2007;(1666):53–68. PubMed PMID: WOS:000251726700004.

[28] Barrett NS. Spotted Handfish Survey. Hobart: CSIRO Division of Fisheries, 1996.

[29] Bruce BD, Green MA. Spotted Handfish Recovery Plan 1999–2001. Hobart: Spotted Handfish Recovery Team, CSIRO Marine Research, 1998.

[30] Commonwealth of Australia. Recovery Plan for Three Handfish Species. Canberra: Department of the Environment, 2015.

[31] Lynch T, Wong L, Green MA. Direct Conservation Actions for the Critical Endangered Spotted Handfish. Hobart: CSIRO Ocean and Atmosphere, 2016.

[32] Bruce BD, Green MA, Last PR. Developing husbandry techniques for spotted handfish (Brachionichthys hirsutus) and monitoring the 1996 spawning season Hobart: CSIRO Division of Marine Research, 1997.

[33] Travis JMJ, Dytham C. Habitat persistence, habitat availability and the evolution of dispersal. P Roy Soc B-Biol Sci. 1999;266(1420):723–8. 10.1098/rspb.1999.0696 PubMed PMID: WOS:000079965900013.

[34] Edgar GJ, Samson CR. Catastrophic decline in mollusc diversity in eastern Tasmania and its concurrence with shellfish fisheries. Conserv Biol. 2004;18(6):1579–88. 10.1111/j.1523-1739.2004.00191.x PubMed PMID: WOS:000225737300021.

[35] Last PR, White WT, Gledhill DC, Hobday AJ, Brown R, Edgar GJ, et al Long-term shifts in abundance and distribution of a temperate fish fauna: a response to climate change and fishing practices. Global Ecology and Biogeography. 2011;20(1):58–72. 10.1111/j.1466-8238.2010.00575.x PubMed PMID: WOS:000285109200005.

[36] Green MA, Bruce BD. Spotted Handfish Recovery Plan 1999–2001: Year 3 Final Report. Hobart: CSIRO Marine Research, 2002.

[37] Green MA. Marine habitat rehabilitation and threatened fish investigation. Hobart: CSIRO Marine and Atmospheric Research, 2005.

[38] Green MA, Stuart-Smith RD, Valentine JP, Einoder LD, Barrett NS, Cooper AT, et al Spotted Handfish monitoring and recovery actions—2011–2012. Hobart: CSIRO Marine and Atmospheric Research/ Institute of Marine and Antarctic Studies, 2012.

[39] Dorenbosch M, van Riel MC, Nagelkerken I, van der Velde G. The relationship of reef fish densities to the proximity of mangrove and seagrass nurseries. Estuar Coast Shelf S. 2004;60(1):37–48. 10.1016/j.ecss.2003.11.018

[40] Zeileis A, Kleiber C, Jackman S. Regression models for count data in R. Journal of Statistical Software. 2008;27(8):1–25. doi: ARTN 8 10.18637/jss.v027.i08 PubMed PMID: WOS:000258207100001.

[41] Anderson TJ, Cochrane GR, Roberts DA, Chezar H, Hatcher G. A Rapid Method to Characterise Seabed Habitats and Associated Macro-organisms In: Todd BJ, Greene HG, editors. Mapping the Seafloor for Habitat Characterisation. SP 47. St. John's: Geological Association of Canada; 2008 p. 71–9.

[42] Green MA, Bruce BD. Spotted Handfish: Distribution, Abundance and Habitat Hobart: CSIRO Division of Marine Research, 1998.

[43] Auster PJ, Lindholm J, Valentine PC. Variation in habitat use by juvenile Acadian redfish, Sebastes fasciatus. Environ Biol Fishes. 2003;68(4):381–9. 10.1023/B:EBFI.0000005751.30906.d5 PubMed PMID: WOS:000186840600007.

[44] Kutti T, Fossa JH, Bergstad OA. Influence of structurally complex benthic habitats on fish distribution. Mar Ecol Prog Ser. 2015;520:175–90. 10.3354/meps11047 PubMed PMID: WOS:000349302600012.

[45] Stoner AW, Spencer ML, Ryer CH. Flatfish-habitat associations in Alaska nursery grounds: Use of continuous video records for multi-scale spatial analysis. Journal of Sea Research. 2007;57(2–3):137–50. 10.1016/j.seares.2006.08.005 PubMed PMID: WOS:000244636400006.

[46] White JW, Samhouri JF, Stier AC, Wormald CL, Hamilton SL, Sandin SA. Synthesizing mechanisms of density dependence in reef fishes: behavior, habitat configuration, and observational scale. Ecology. 2010;91(7):1949–61. . 20715614 10.1890/09-0298.1

[47] Jordan F, Bartolini M, Nelson C, Patterson PE, Soulen HL. Risk of predation affects habitat selection by the pinfish Lagodon rhomboides (Linnaeus). J Exp Mar Biol Ecol. 1997;208(1–2):45–56. 10.1016/S0022-0981(96)02656-1 PubMed PMID: WOS:A1997WC65300004.

[48] Swearer SE, Shima JS, Hellberg ME, Thorrold SR, Jones GP, Robertson DR, et al Evidence of self-recruitment in demersal marine populations. B Mar Sci. 2002;70(1):251–71. PubMed PMID: WOS:000176377500002.

[49] Strathmann RR, Hughes TP, Kuris AM, Lindeman KC, Morgan SG, Pandolfi JM, et al Evolution of local recruitment and its consequences for marine populations. B Mar Sci. 2002;70(1):377–96. PubMed PMID: WOS:000176377500007.

[50] Saucier MH, Baltz DM. Spawning site selection by spotted seatrout,Cynoscion nebulosus, and black drum,Pogonias cromis, in Louisiana. Environ Biol Fishes. 1993;36(3):257–72. 10.1007/bf00001722

[51] Moriarty T. Can a Spotted Handfish (Brachionichthys hirsutus) change its spots? Assessing photo-identification and spot matching software to study a critically endangered species [BMarSc (Hons) thesis]. Hobart: University of Tasmania; 2012.

[52] Courchamp F, Clutton-Brock T, Grenfell B. Inverse density dependence and the Allee effect. Trends in Ecology & Evolution. 1999;14(10):405–10. 10.1016/S0169-5347(99)01683-3 10481205

[53] Gascoigne J, Berec L, Gregory S, Courchamp F. Dangerously few liaisons: a review of mate-finding Allee effects. Popul Ecol. 2009;51(3):355–72. 10.1007/s10144-009-0146-4 PubMed PMID: WOS:000266486500003.

[54] Gascoigne J, Lipcius RN. Allee effects in marine systems. Mar Ecol Prog Ser. 2004;269:49–59. 10.3354/meps269049 PubMed PMID: WOS:000221060100004.

[55] Arroyo NL, Aarnio K, Maensivu M, Bonsdorff E. Drifting filamentous algal mats disturb sediment fauna: Impacts on macro-meiofaunal interactions. J Exp Mar Biol Ecol. 2012;420:77–90. 10.1016/j.jembe.2012.03.020 PubMed PMID: WOS:000305260200010.

[56] Lucieer VL, Lawler M, Morffew M, Pender A. Estuarine Habitat Mapping in the Derwent—2007 A Resurvey of Marine Habitats by SeaMap Tasmania Hobart: Tasmanian Aquaculture and Fisheries Institute, University of Tasmania, 2007.

[57] Byrne M, Morrice MG, Wolf B. Introduction of the northern Pacific asteroid Asterias amurensis to Tasmania: Reproduction and current distribution. Marine Biology. 1997;127(4):673–85. 10.1007/s002270050058 PubMed PMID: WOS:A1997WQ42500017.

[58] Ross DJ, Johnson CR, Hewitt CL. Impact of introduced seastars Asterias amurensis on survivorship of juvenile commercial bivalves Fulvia tenuicostata. Mar Ecol Prog Ser. 2002;241:99–112. 10.3354/meps241099 PubMed PMID: WOS:000179145900009.

[59] Auster PJ, Malatesta RJ, Langton RW, Watting L, Valentine PC, Donaldson CLS, et al The impacts of mobile fishing gear on seafloor habitats in the gulf of Maine (Northwest Atlantic): Implications for conservation of fish populations. Reviews in Fisheries Science. 1996;4(2):185–202. 10.1080/10641269609388584

[60] Scharf FS, Manderson JP, Fabrizio MC. The effects of seafloor habitat complexity on survival of juvenile fishes: Species-specific interactions with structural refuge. J Exp Mar Biol Ecol. 2006;335(2):167–76. 10.1016/j.jembe.2006.03.018 PubMed PMID: WOS:000239232500002.

[61] Lande R. Demographic stochasticity and Allee effect on a scale with isotropic noise. Oikos. 1998;83(2):353–8. 10.2307/3546849 PubMed PMID: WOS:000076942100018.

[62] MAST. MAST Mooring Review 2016. Hobart: Marine and Safety Tasmania, 2016.

[63] DoE. Recovery Plan for Three Handfish Species. Canberra: Department of the Environment, 2015.

[64] Baguette M, Schtickzelle N. Local population dynamics are important to the conservation of metapopulations in highly fragmented landscapes. J Appl Ecol. 2003;40(2):404–12. 10.1046/j.1365-2664.2003.00791.x PubMed PMID: WOS:000182118700018.

[65] Joseph LN, Field SA, Wilcox C, Possingham HP. Presence–Absence versus abundance data for monitoring threatened species. Conserv Biol. 2006;20(6):1679–87. 10.1111/j.1523-1739.2006.00529.x 17181803

