# Correction: Local densities and habitat preference of the critically endangered spotted handfish (*Brachionichthys hirsutus*): Large scale field trial of GPS parameterised underwater visual census and diver attached camera

**DOI:** 10.1371/journal.pone.0205040

**Published:** 2018-09-26

**Authors:** The PLOS ONE Staff

The following information is missing from the Funding section: This work was partly funded by the Marine Biodiversity Hub, a collaborative partnership supported through funding from the Australian Government's National Environmental Science Programme. The funding body was not involved in the process of study design, data collection and analysis, decision to publish, or preparation of the manuscript.

There is an error in the Acknowledgments section. The name "Ashley Leeman" is spelled incorrectly. The correct spelling is: Ashley Leedman.
